# Spatial Distribution of Oak Mistletoe as It Relates to Habits of Oak Woodland Frugivores

**DOI:** 10.1371/journal.pone.0111947

**Published:** 2014-11-12

**Authors:** Ethan A. Wilson, Patrick J. Sullivan, Janis L. Dickinson

**Affiliations:** 1 Department of Natural Resources, Cornell University, Ithaca, New York, 14850, United States of America; 2 The Cornell Lab of Ornithology, 159 Sapsucker Woods Road, Ithaca, New York, 14850, United States of America; College of Charleston, United States of America

## Abstract

This study addresses the underlying spatial distribution of oak mistletoe, *Phoradendron villosum*, a hemi-parasitic plant that provides a continuous supply of berries for frugivorous birds overwintering the oak savanna habitat of California's outer coast range. As the winter community of birds consuming oak mistletoe varies from group-living territorial species to birds that roam in flocks, we asked if mistletoe volume was spatially autocorrelated at the scale of persistent territories or whether the patterns predicted by long-term territory use by western bluebirds are overcome by seed dispersal by more mobile bird species. The abundance of mistletoe was mapped on trees within a 700 ha study site in Carmel Valley, California. Spatial autocorrelation of mistletoe *volume* was analyzed using the variogram method and spatial distribution of oak mistletoe *trees* was analyzed using Ripley's K and O-ring statistics. On a separate set of 45 trees, mistletoe volume was highly correlated with the volume of female, fruit-bearing plants, indicating that overall mistletoe volume is a good predictor of fruit availability. Variogram analysis showed that mistletoe volume was spatially autocorrelated up to approximately 250 m, a distance consistent with persistent territoriality of western bluebirds and philopatry of sons, which often breed next door to their parents and are more likely to remain home when their parents have abundant mistletoe. Using Ripley's K and O-ring analyses, we showed that mistletoe trees were aggregated for distances up to 558 m, but for distances between 558 to 724 m the O-ring analysis deviated from Ripley's K in showing repulsion rather than aggregation. While trees with mistletoe were aggregated at larger distances, mistletoe was spatially correlated at a smaller distance, consistent with what is expected based on persistent group territoriality of western bluebirds in winter and the extreme philopatry of their sons.

## Introduction

The spatial distribution of resources is an important driver of social behavior in a range of animal species [1], [2], [3]. Where food plants and animal social systems are interdependent, they can become mutual engineers of each other's demography, dispersal, distribution, and abundance. Here we examine the spatial distribution of a key winter resource, oak mistletoe *(Phoradendron villosum*), in light of the behavior of its winter frugivores, which vary from species that hold persistent year-round territories to those that flock and adopt a mobile lifestyle in winter. Mistletoes are recognized as keystone resources throughout the world [4].

Multi-scalar assessment of landscape patterns is an important precursor to understanding the distinct processes that give rise to ecological patterns [5], but intensive data collection on keystone species is often lacking. Group-territorial frugivores like western bluebirds (*Sialia mexicana*) are likely to disperse seeds locally, producing a clumping of resources at the scale of one to two territories, whereas mobile species will tend to disperse seeds more broadly, leading to aggregation of mistletoe over larger areas. From the point of view of the plants, territorial frugivores may reduce opportunities for long distance dispersal of seeds [6]. Here we conduct spatial analysis at two different scales to look for signatures consistent with both types of seed dispersers.

Western bluebirds are common in the outer coast range of California, occupying persistent year-round territories and regularly overwintering in family groups after a brief period of juvenile dispersal in late summer and early fall [7]. The ecological driver of year-round territoriality and family-group living in winter is oak mistletoe, which is hemiparasitic on certain species of oak trees [8], [9]. Mistletoe plants produce a large and constant supply of berries from October until early spring, providing a stable winter food resource for western bluebird family groups (mistletoe seeds appear in 100% of fecal samples in winter, [10].) Western bluebirds in turn act as local dispersal agents for mistletoe plants through continuous, multi-generational occupancy of year-round territories, frequent use of favorite perches, and viscous patterns of settlement in which sons nest near their parents [11], [12].

The importance of mistletoe to delayed dispersal of western bluebirds was established at Hastings Reserve in Carmel Valley, CA with an experiment in which half the mistletoe was removed from a set of territories. Only 10% of removal territories retained sons through their first winter, compared to 87% of control territories. When parents left their territories for winter, their territories had lower volumes of mistletoe than did territories of parents remaining for winter [10]. While this experiment demonstrated that mistletoe is a causal agent for philopatry of sons [10], direct examination of the influence of western bluebirds on the dispersal and establishment of mistletoe is more challenging, because oak mistletoe germinates rarely, grows slowly, and persists on trees in excess of 60 years, based on a collection of photographs from the Hastings Reserve housed in the Museum of Vertebrate Zoology, UC Berkeley. Given that sons born on high quality territories tend to remain home and form kin neighborhoods in the vicinity of their parents, areas with abundant mistletoe tend to be used persistently. This should augment the “planting” side of the interaction producing pockets of high quality territories and thus spatial autocorrelation of mistletoe abundance, which we measure as total volume per tree. Thus spatial autocorrelation of mistletoe volume may both augment the benefits of delayed and localized dispersal in western bluebirds and also be a direct consequence of this augmentation via a positive feedback loop.

Other common winter frugivores at Hastings Reserve include American robins, *Turdus Migratorius*, and cedar waxwings, *Bombycilla cedrorens*. Where they have been studied, American robins can be territorial in winter, eat a wide variety of fruits, and engage in fruit defense against both hetero- and conspecifics [13]. Juveniles robins sometimes remain in flocks near their natal territories and these flocks remain until food supplies become insufficient [14], [15]. Mobile feeding flocks of more than 250 American robins are also seen, indicating plasticity depending on the distribution of resources and on the ability of group size to increase success competing for resources [15], [16]. Cedar waxwings, *Bombycilla cedrorum*, occur commonly at Hastings Reserve. They are nonterritorial throughout the year and, although their nests are aggregated in spring, they flock to and aggregate at fruiting sites away from the nest [17]. Their diets are comprised almost entirely of fruit in winter, and they eat a variety of fruit species as they move about in roving flocks [18]. Other frugivore species appear less important, such as band-tailed pigeons, *Patagioenas fasciata*, which are not known to be territorial in winter and commonly eat acorns in addition to other fruits [19]. Two territorial frugivores, Townsend's solitaires, *Myadestes townsendi*, and Phainopeplas, *Phainopepla nitens*, are rare in the outer coast range of California. If western bluebird territoriality concentrates dispersal of mistletoe within and around territories, mistletoe abundance will be spatially autocorrelated at the scale of territories; on the other hand, patterns of spatial autocorrelation may be disrupted by longer distance dispersal of seeds, for example, by roving flocks of species such as cedar waxwings, which are also common at Hastings Reserve.

In this paper we address the potential for multi-scalar patterns of abundance and occurrence of oak mistletoe, based on differences in seed dispersal distances for territorial versus the more mobile frugivores feeding on oak mistletoe. Using the variogram method we determine whether spatial autocorrelation of mistletoe *volume* per tree occurs over distances consistent with prolonged territory occupancy, winter family group living, territory budding, and localized dispersal of western bluebird sons. We then use Ripley's K and O-ring statistics to ask over what distance trees with mistletoe are spatially aggregated. Our expectation is that spatial autocorrelation resulting from localized dispersal of mistletoe seeds by western bluebirds will be apparent within a background of larger scale aggregation of oak trees infested with mistletoe.

## Methods

### Study areas

The mistletoe data used in this paper were collected in conjunction with a long-term (1983-present) study of box-nesting, color-banded western bluebirds breeding and wintering on Hastings Reserve and the contiguous Oak Ridge Ranch, Carmel Valley, California (herein referred to as Hastings/Oak Ridge). Identical monitoring procedures were also instigated on nearby Rana Creek Ranch from 2001–2006. Data on dispersal distances of marked natal group males were collected from Hastings/Oak Ridge and Rana Creek Ranch (2001–2006).

### Relative frequency of three main frugivores

The relative frequency of the three main frugivores was estimated using publicly available data from eBird observations entered from 2006–2014 on our study area [20]. We only considered counts conducted from September through February, eliminating redundant entries in cases where more than one individual reported data on the same birding trip.

### Mistletoe data collection

Data on mistletoe-parasitized trees were collected during the years 2001–2003 by traversing the area within 200 m of each of 363 nest boxes on the contiguous Hastings/Oak Ridge site. Our study site is well north of Arroyo Grande, CA, the dividing line above which oak mistletoe grows mostly on blue oak and valley oak trees (*Quercus douglasii* and *Quercus lobata*, respectively) and only rarely on coast live oak (*Quercus agrifolia*) [21]. Data were collected after leaf drop and before trees had fully leafed out again. The Hastings/Oak Ridge trees (n = 2,658) were geolocated using a differential global positioning system (Garmin), marked with uniquely numbered aluminum tree tags, and photographed next to a reference stake using a digital camera. The number of mistletoe clumps was counted and classified in the field for each tree using 6 size categories (diameter <20, 20–50, 50–75, 75–100 cm; 1–1.5 m). Mistletoe volume was calculated for the first five categories using the formula for the volume of a sphere with radius 10, 20, 35, 60, and 75 cm and summed to obtain a total mistletoe volume for each tree. Clumps measuring>1.5 m tended to be elongate and their volume was approximated as the sum of the volume for two or more 75 cm diameter spheres [10]. Data were compiled and organized within ArcGIS10 [22], and analyzed using statistical software package R [23].

In addition to collecting data on mistletoe as described above, we selected a subset of trees from another of our nearby study plots (Rana Creek Ranch) to investigate the relationship between mistletoe volume and the volume of female, fruit-bearing mistletoe plants; *P. villosum* is dioecious. In October - December of 2001 we selected and photographed a sample of 45 mistletoe-bearing blue oaks (*Quercus douglasii*) and valley oaks (*Quercus lobata*). Close inspection of trees in the field revealed that large clumps of mistletoe were often aggregates of a set of smaller clumps whose exact number was not possible to estimate. All accessible mistletoe clumps with flowers and fruits were sexed during routine, weekly phenology assessments. We used a Pearson correlation to assess the relationship between total clump volume per tree (which could be measured for all trees in our study), and total female clump volume per tree, which was feasible to measure only for a small number of trees. These measures served to test the appropriateness of using total mistletoe volume as a proxy for volume of female, fruit-bearing mistletoe available as food for western bluebirds.

### Winter territory size, dispersal distance, and spring retention of philopatric sons

Location data for assessing western bluebird territory size were collected in winter of 2004–5 from the Hastings/Oak Ridge and Rana Creek sites. Winter groups were censused for the presence of banded birds (and to band any unbanded individuals) from September through February in 2001–2006. Groups were stable by mid-October. During winter censuses, we recorded identities of all birds in the group and recorded locations to construct territory maps. We used methods similar to those described in Dickinson and McGowan [10], creating maps based upon independent data points gathered during repeat censuses of winter territories. Territories were assumed to encompass the area covered by the minimum convex polygon formed by the mapped points. In order to obtain a proxy for the bluebirds' radial territory extent, we used the total area for each territory map and computed the associated radius of a circle having the same area. The mean of these measures could then be compared to the degree of spatial dependence of mistletoe volume, as determined by the fitted variogram (see below).

To ask whether mistletoe volume was higher on territories where sons stayed home for winter and also remained on the study area in spring, we used a generalized linear model (Poisson distribution) with presence or absence in spring as a fixed factor, year as a random factor, and mistletoe volume within 100 m of the natal nest box as the response variable. The volume of mistletoe within 100 m of the natal nest box was previously shown to be positively correlated with the volume within 100 m of the central nest box on the winter territory, which in turn was positively correlated with the volume on 10 territories that were extensively mapped [9]. This data set included all sons remaining on their natal territories in winter for the five-year period where we had year-round data. Considering only the 166 males that remained in their natal groups for winter, we compared the natal territories of those that remained on the study area the following spring with natal territories of those that disappeared after surviving the winter.

In order to evaluate the dispersal distances of philopatric sons, we examined the distance dispersed by 82 males that remained on their natal territories their first winter and subsequently bred on the study area as yearlings. This analysis included winter groups monitored in 2001–2006 at Hastings Reserve and Rana Creek Ranch, and allowed us to assess the extent of localized dispersal for males that remained in their natal groups for winter [7].

### Vegetation layer describing blue oak-valley oak woodland

Over 99% of the oak mistletoe trees on our study area are blue oaks or valley oaks. Starting with vegetation classifications based on remote sensing data, we used TNTmips from 60 cm RGB and CIR aerial imagery to generate an improved vegetation map classifying the four main landcover types of interest (chaparral, blue oak-valley oak woodland, mixed oak-madrone forest, and open savanna grassland). The original remote sensing vegetation classifications more fine-grained and were found to be inaccurate at distinguishing the major vegetation types of interest; an improved classification of the vegetation layer was derived using Manifold v6.5 (Manifold Net Ltd., Carson City, NV, USA) using majority pixel analysis, which calculates the majority values over a windowed area to deal with remaining errant vegetation areas. Errors due to shadow effects were then corrected via groundtruthing and comparison to high resolution aerial photos, using the combined expertise of two individuals (J.L. Dickinson and Walter D. Koenig) who worked and lived year-round on the study area for more than 18 and 31 years, respectively.

### Spatial autocorrelation of mistletoe volume

We then examined the spatial autocorrelation of the continuous variable, mistletoe *volume*, in order to test for evidence consistent with the hypothesis that sons remaining on high mistletoe volume territories *and* establishing territories adjacent to their parents' territories will have an increased chance of acquiring a high mistletoe volume territory themselves (spatial dependence for all distances less than mean winter radius plus some distance representative of territories immediately surrounding the parents' territories). Alternatively, if no spatial autocorrelation exists, this would suggest that the mistletoe volume on a male's natal territory is a poor predictor of what he can expect to acquire by staying home for winter and dispersing next door to his parents. Spatial autocorrelation of mistletoe volume at a larger scale would not be consistent with expectations based on persistent group territoriality of western bluebirds.

**Figure 1:**
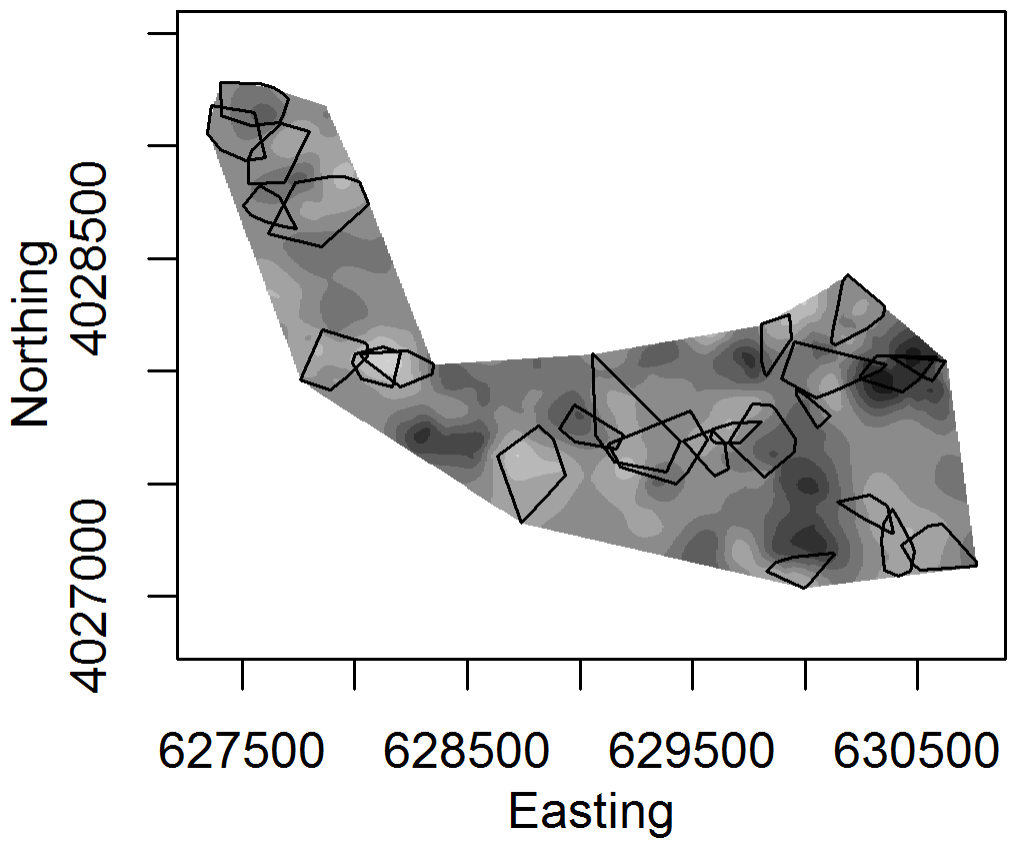
Kriged surface image of predicted mistletoe log-volume, overlaid by mapped winter territories (2004–2005, from a portion of the larger study area) and the minimum bounding convex polygon for all territories. White color represents high concentrations of mistletoe volume, while dark areas are indicative of lower intensity mistletoe volume.

In order to analyze the spatial autocorrelation of mistletoe, we used a log-transform of the volume measure. Simulation of the observed process underlying mistletoe volume was modeled by fitting a spherical variogram to the sample variogram. Due to the finite range of points, the sample variogram may contain a signal due to sampling error. To test for the significance of spatial autocorrelation via the variogram, we ran 1000 simulations in which the measurements for log-volume were randomly re-assigned to the mistletoe data point locations, while the locations themselves remained the same. If the observed sample variogram falls within the 95% range of these simulated variograms, we then conclude that any autocorrelation may have occurred by chance [24]. Variogram analyses were performed in R, using the package gstat [25]. Using the fitted variogram, we created a kriged surface image portraying spatially interpolated measures of mistletoe log-volume (Fig. 1). Winter territory maps for western bluebirds were then overlaid on this image to examine territory placement with respect to mistletoe abundance.

### Spatial Point Pattern analysis for spatial aggregation of mistletoe-bearing trees

To measure the degree of spatial aggregation of the trees bearing mistletoe, we employed the Ripley's K-function and the O-ring statistic, complementary spatial statistical methods used to quantify aggregation in spatial point patterns [26], [27]. The Ripley's K statistic is based on the accumulation of points within a circle of increasing radius, while the O-ring statistic considers points within an annulus of increasing size. This distinction implies that as the scale of aggregation increases, strong small scale aggregation effects will continue to influence measures of large scale aggregation in the Ripley's K analysis, but not in the O-ring analysis [27].

**Figure 2:**
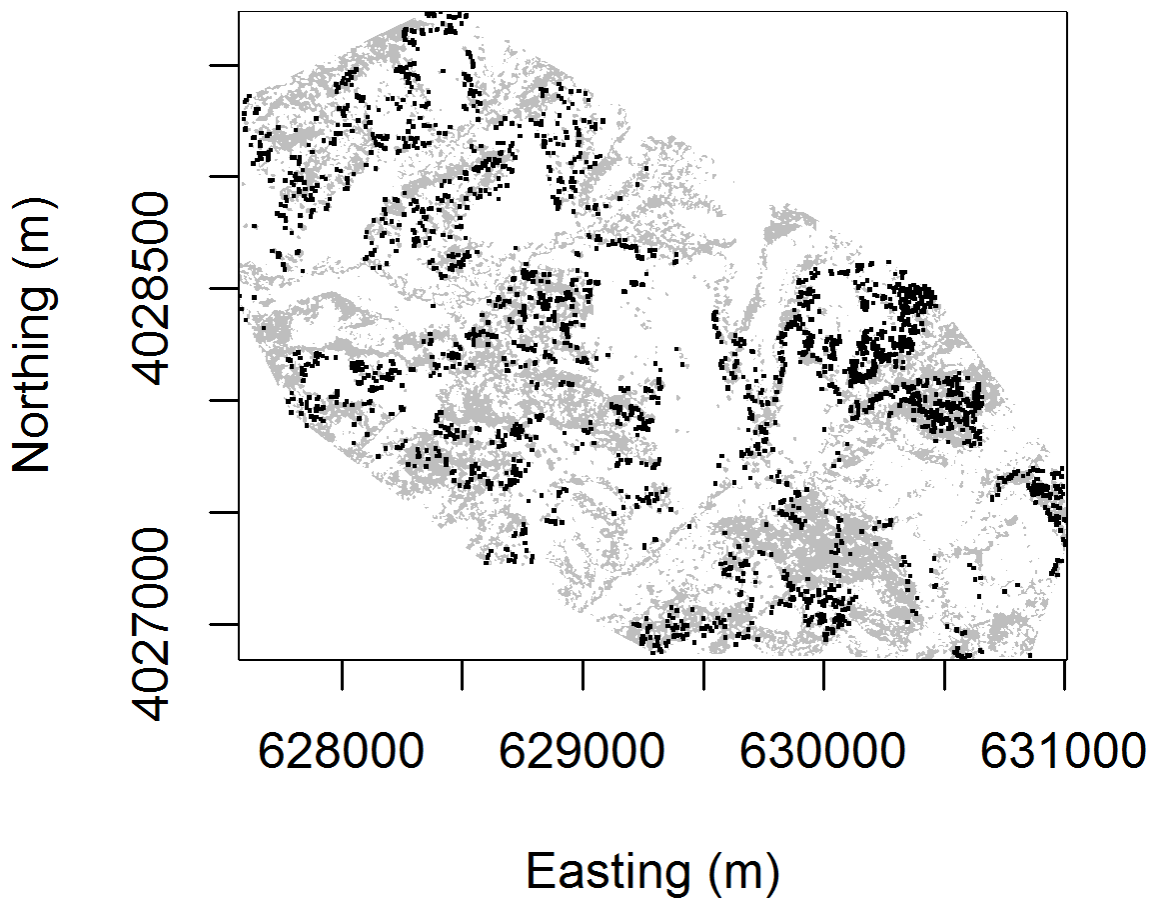
Distribution of mistletoe points (black) on Hastings/Oak Ridge within the extent of our study site. The oak background (grey) was defined spatially as the convex hull derived from the mistletoe points.

Spatial aggregation patterns were analyzed for Hastings/Oak Ridge for the total mistletoe-bearing tree population (n = 2658 trees). Beginning with a vegetation map of blue and valley oak woodland (Fig. 2), we assumed based on our data that mistletoe growth only occurs within the real extent of blue oak-valley oak woodland. Our null model for the spatial extent of mistletoe habitat was thus restricted to that of its local host species, blue and valley oaks, as recommended in previous ecological literature [28], [29].

In both the Ripley's K and O-ring analyses, Monte Carlo simulations (199 total) were used to test the null hypothesis that the mistletoe points (trees) were distributed according to a homogeneous Poisson process. We did this by randomly reallocating 2658 points (trees), restricted to the spatial extent of oak vegetation. For each simulation, a test statistic was derived yielding a range of estimates for the expected value of the statistic under the null hypothesis. Using the results of these simulations, we derived approximate 95% confidence envelopes by using the 5^th^ lowest and highest of the simulated values for the statistic. These simulations were then compared to the observed test statistic for the true distribution of mistletoe points. The Ripley's K analysis was performed in R, using the package spatstat [30] and the O-ring analysis was performed using the software package Programita [27].

### Ethics Statement

The study was conducted on Hastings Natural History Reservation, a University of California Reserve associated with UC Berkeley (the UTM coordinates of Hastings office: Easting: 630032 Northing: 4027902). Queries to conduct research on this site should be communicated through the reserve director, who is also the broker for any work done on private lands adjacent to the reserve.

The work was conducted on USFWS banding permit # 23533 to Janis L. Dickinson and California Fish and Game permit # 801053 to Janis L. Dickinson. The field studies did not involve endangered or protected species. No animals were sacrificed or collected. For the purposes of this study, western bluebirds were color-banded and observed for 1-2 hours at a time to map territories. Animal use protocols are described in IACUC AUP #R212 at UC Berkeley and IACUC #2005–0137 at Cornell University.

## Results

### Frequency of three main frugivores on study area

Based on 17 independent eBird counts collected over 8 winters from 2006–2014, the frequency of these three species was: 57% western bluebirds; 21% cedar waxwings; and 22% American Robin. The maximum number of individuals counted at one time for each species was: 12 western bluebirds, 15 cedar waxwings, and 12 American Robins.

### Mistletoe volume as a valid proxy for female mistletoe volume

**Figure 3:**
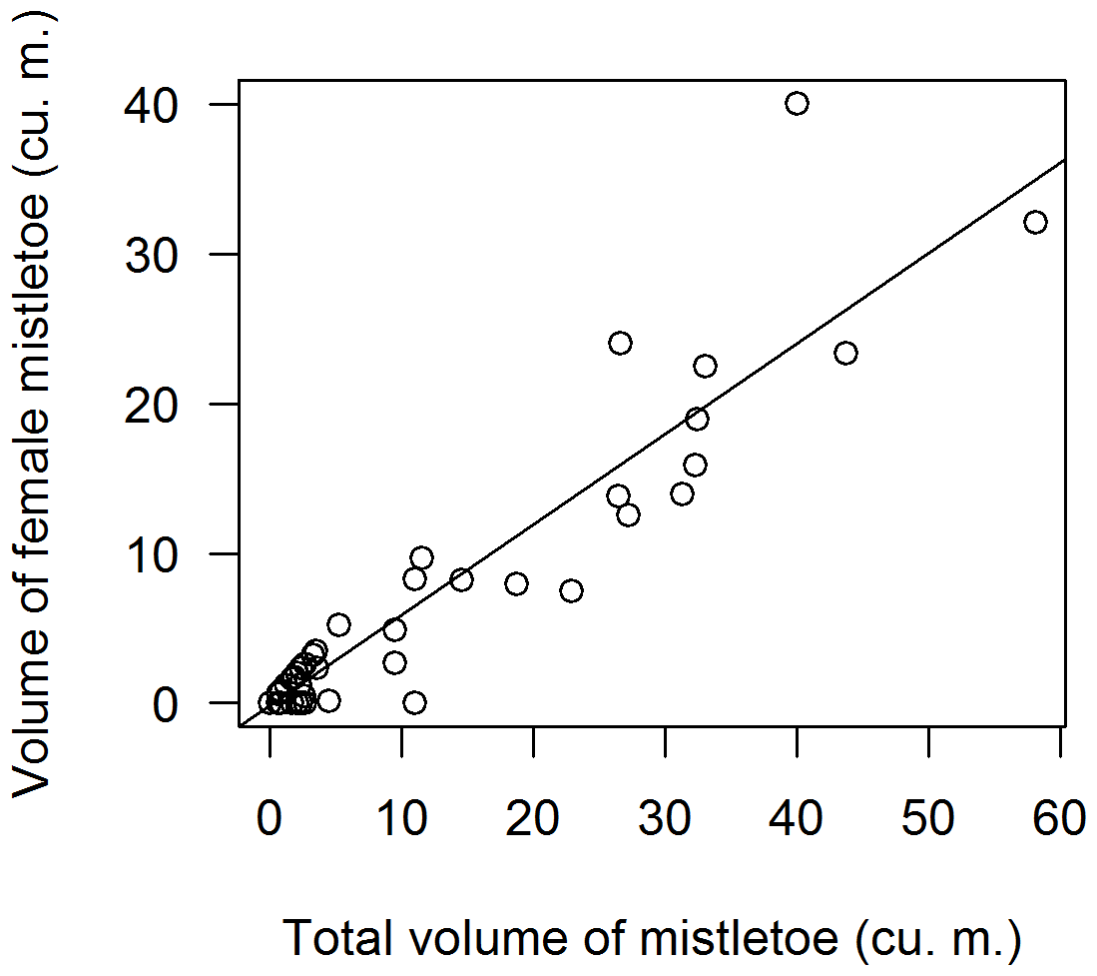
Female mistletoe volume correlated with total mistletoe volume (*Phoradendron villosum*) on 45 blue oak and valley oak trees (*Quercus douglasii* and *Q. lobata*, respectively) monitored for berry production over the winter of 2001–2002 (r = 0.92, P<0.001).

In order to determine whether total volume per tree is a good proxy for the volume of female (fruiting) clumps per tree, we compared total and female mistletoe volume for 45 trees. Female volume increased linearly with total volume (*r* ^2^ = 0.86, *F*_1,43_ = 265, *P*<0.001, Fig. 3).

### Territory size for wintering western bluebirds

Extensive mapping of western bluebird territories in winter 2004–5 resulted in 25 territory maps for analysis on Hastings/Oak Ridge. The mean area of these territories was 41663±5062 m^2^, corresponding to a circular radius of 115 m. By finding the radius of a circle having the estimated mean area, we obtained an approximation of the distance from the center of a territory to its boundary, given that territories are not truly circular in shape. This distance was used in order to make inferences about the relationship between dispersal distance from territories and distance of spatial autocorrelation of mistletoe volume.

### Western bluebird sons' dispersal distances after winter

**Figure 4:**
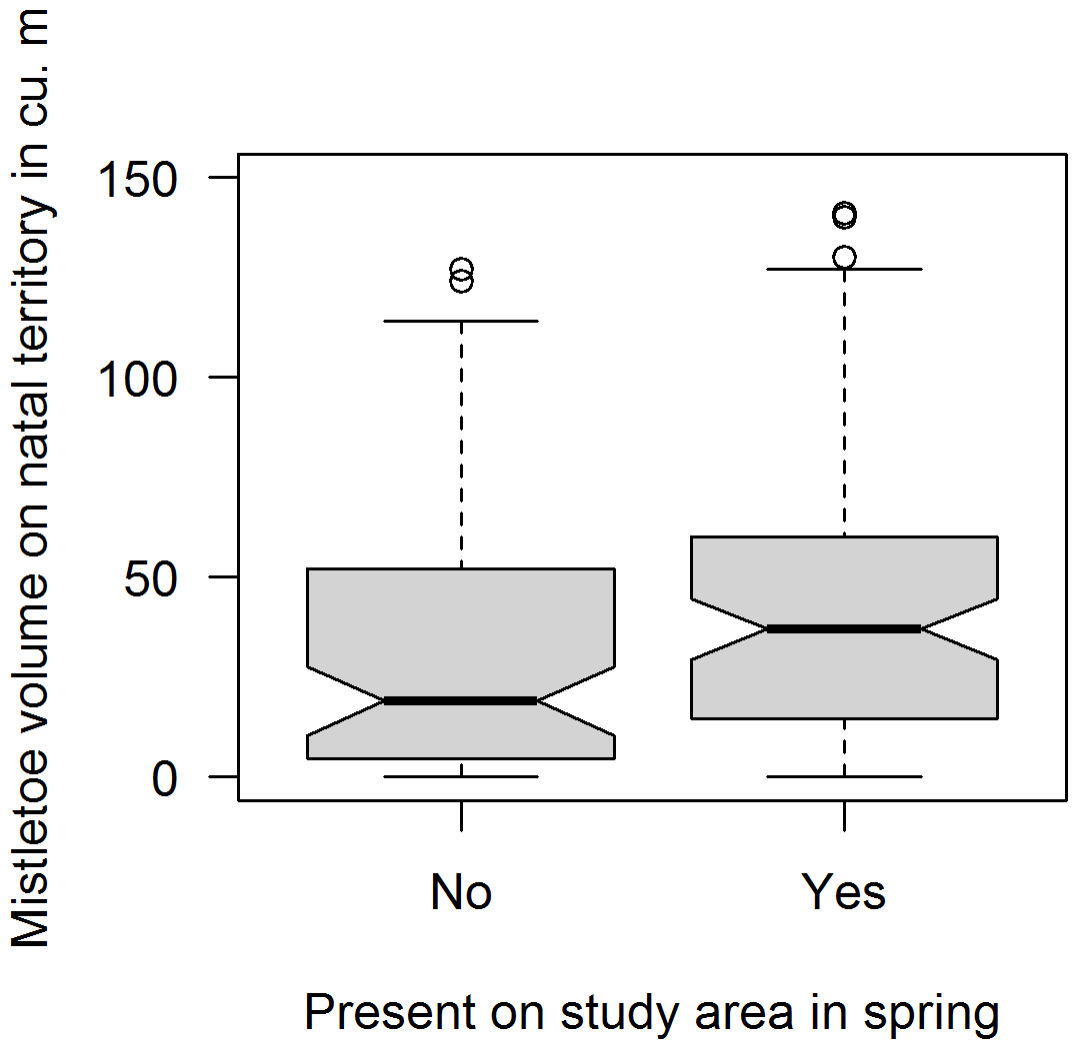
Box plots demonstrating that for first-winter male western bluebirds wintering in their natal groups and surviving the winter, those present on the study area in spring had higher mistletoe volume within 100 m of their natal nest box than did those not present in spring.

**Figure 5:**
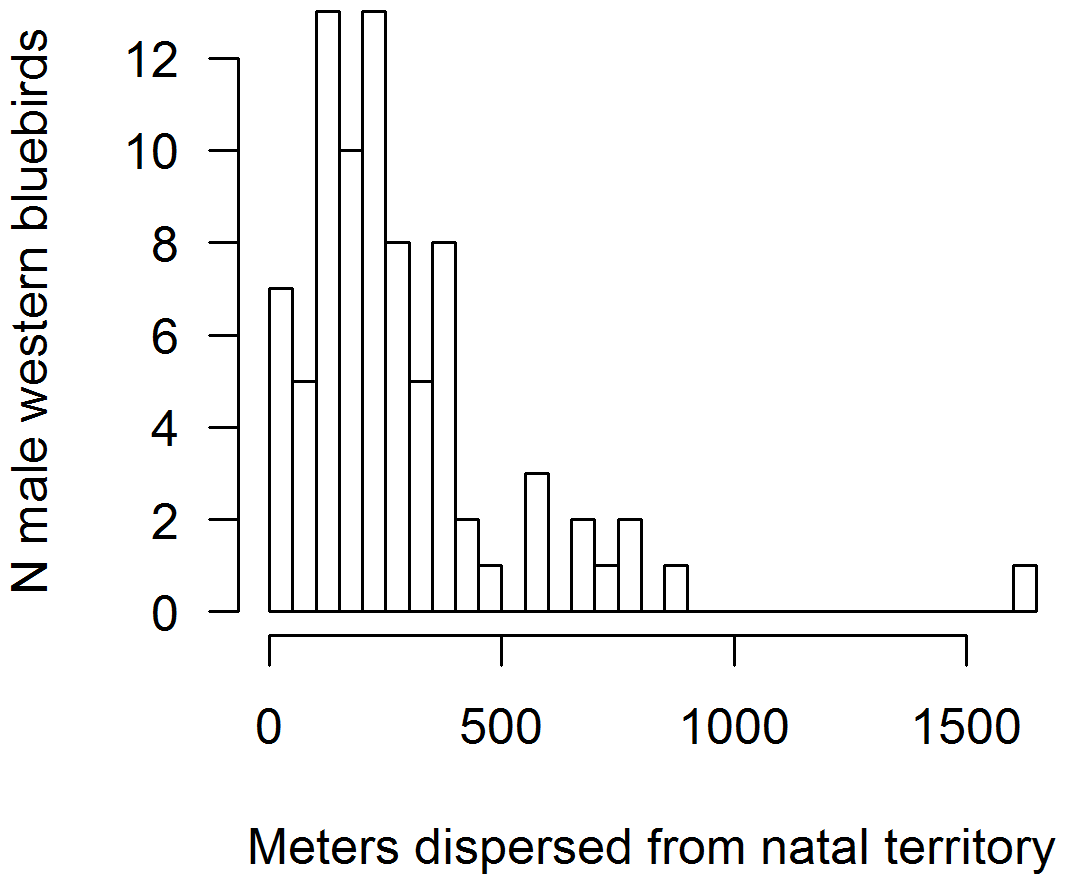
Histogram of dispersal distances for western bluebird males (*Sialia Mexicana*) that wintered in their natal group in winter of 2001–2006 and bred on the study area the subsequent spring.

Males that settled on the study area in the breeding season came from territories with higher volumes of mistletoe than those that dispersed away from the study area after winter (lmer, mistletoe volume: *z* = 13.0, N = 166 males, *P*<0.001 (year as random factor), Fig. 4). Fig. 5 shows the combined within-site dispersal distribution for males that remained on their natal territories for winter at Hastings/Oak Ridge and Rana Creek Ranch sites. Approximately 99% of 82 recovered natal sons that bred settled <900 m from their natal box and 58.5% bred within 250 m from their natal nest (Fig. 5), indicating that over half of sons that remained on their natal territory for winter bred within one territory of their natal nest. We detected one long-distance dispersal of a male about 5.6 km between the Hastings/Oak Ridge and the Rana Creek Site (not included in Fig. 5, nor in other analyses).

### Spatial autocorrelation of mistletoe volume

**Figure 6:**
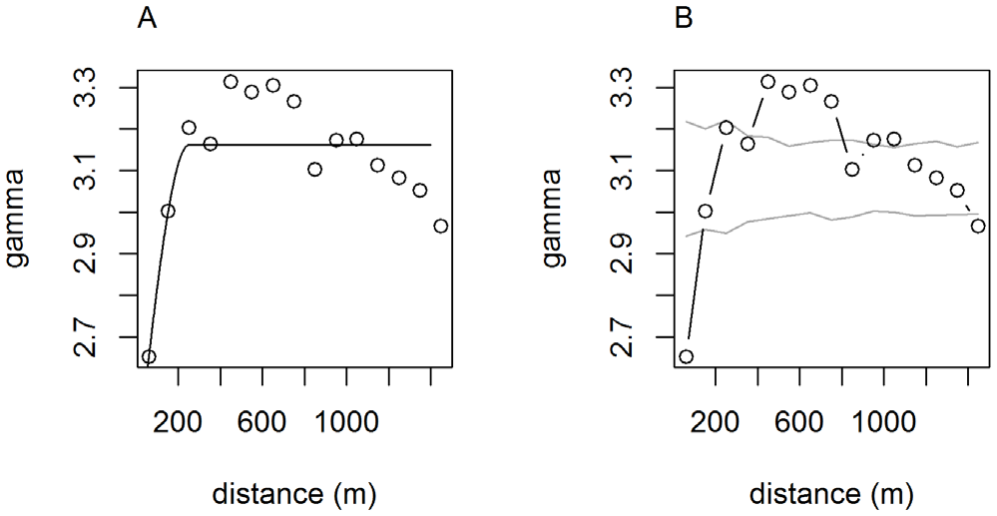
Points and line representing the empirical and fitted variogram, respectively. Parameters for the fitted spherical variogram include: partial sill  = 0.79, nugget  = 2.4 and range  = 250.3 m (A). Permutation test of the variogram, which determines the general range of values that could occur due to random chance. The black line represents the true variogram, and the gray lines represent a 95% confidence envelope of 1000 variogram simulations of randomly permuted mistletoe volume measures; each using the same point locations as the original data (B).

Mistletoe volume was analyzed using the fitted and empirical variograms. The variogram plots the semi-variance of the response variable, log-volume, as a function of distance. Plots of these, along with the parameter values for the nugget, partial sill, and range of the fitted spherical model, are represented in Fig. 6. The variogram range parameter is interpreted as evidence of spatial autocorrelation of mistletoe volume for all distances <250 meters. Fig. 6 also shows the sample variogram, compared to 1000 variograms of randomly reallocated data. The permutation test indicates that the hypothesis of absence of spatial autocorrelation of mistletoe is unlikely, as much of the empirical variogram lies outside of the major range of the permuted values [24]. Overlaying winter territory boundaries onto a kriged surface of mistletoe volume shows visually how remaining on territories with high levels of mistletoe abundance provides opportunities for acquisition of high quality territories nearby (Fig. 1).

### Spatial aggregation measure

**Figure 7:**
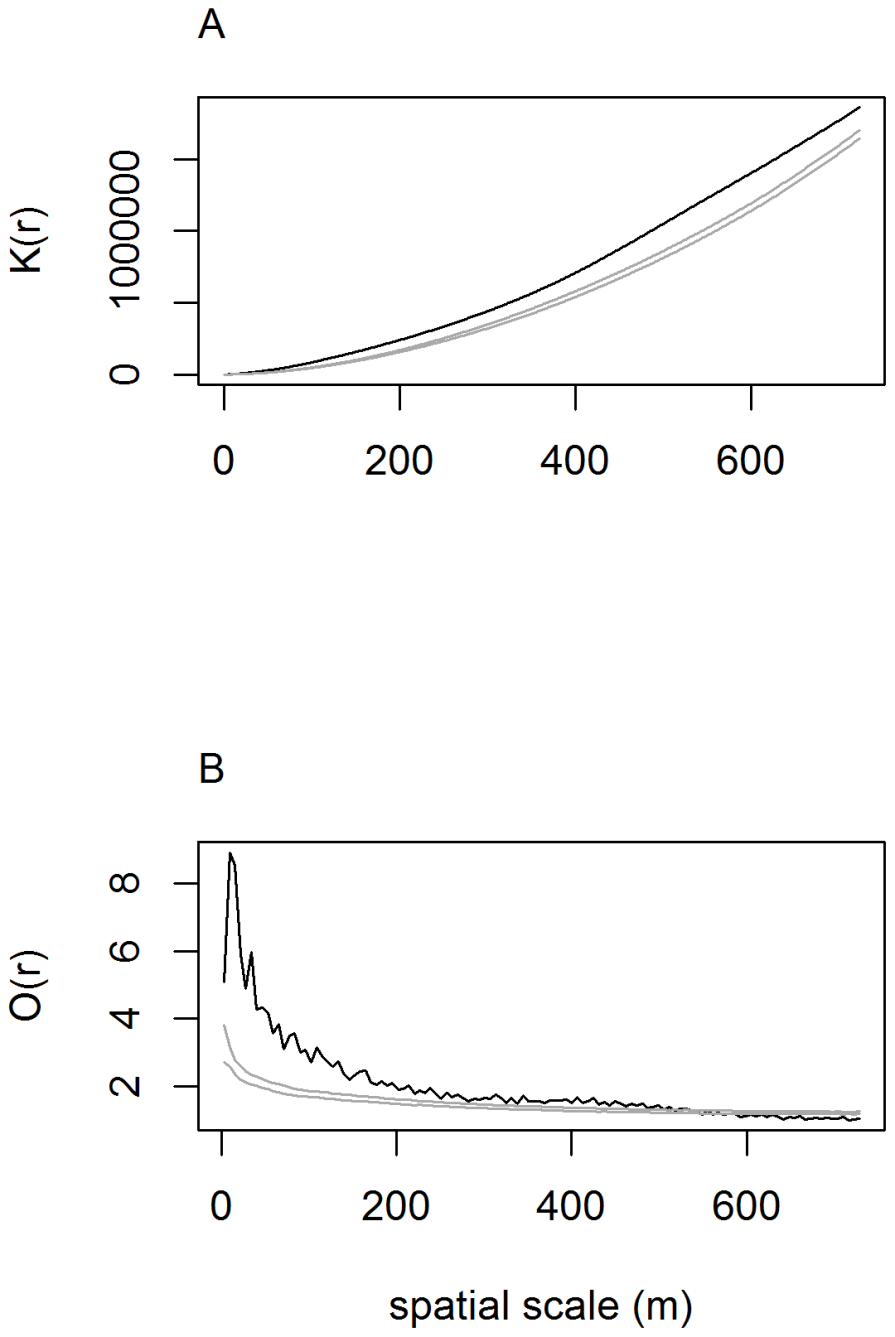
Graph of the observed Ripley's K-statistic for the true distribution (black line), showing the average number of trees parasitized with mistletoe within a given distance. The gray lines represent the 95% confidence envelopes for complete spatial randomness, based on 199 Monte Carlo simulations of 2658 random data points within the oak vegetation area (A). Graph of the observed O-ring statistic for the true distribution (black dotted line), as well as the 95% confidence envelopes (gray lines) for complete spatial randomness. This analysis was performed using a cell size of 6.2 m^2^ and a ring width of 18.6 m. Both analyses were restricted to a maximum distance of 724 m (B).

Both the Ripley's *K*-function and the O-ring statistic demonstrated strong aggregation of mistletoe-bearing trees for distances up to 558 meters. However, for distances between 558 to 724 meters the Ripley's K analysis showed evidence of aggregation, while the O-ring analysis showed evidence of dispersion. The graph in Fig. 7 shows that for all distances measured, the observed K statistic lies above the confidence envelope, indicative of spatial aggregation, while the O-ring statistic transitions from above to below the confidence envelop at about 558 m. Although the graph of the observed O-ring statistic does not deviate far below the confidence envelope, it provides evidence of spatial repulsion that was not detected by the Ripley's K analysis.

## Discussion

Analysis of the variogram indicates that mistletoe volume is spatially autocorrelated for distances less than 250 meters. Given that it is only feasible for mistletoe to occur within the extent of blue and valley oak vegetation, spatial autocorrelation of volume up to 250 meters is consistent with the extent of western bluebirds' winter territories. This finding is in accordance with Aukema's (2004) suggestion that positive feedbacks can result in mistletoe augmentation at the scale of small neighborhoods by territorial bird species, although our focus here is on winter territoriality and winter fruit production [5]. As Fig. 1 indicates, from a western bluebird's point of view, the distribution of mistletoe wealth is heterogeneous at scales relevant to clustering of families on high mistletoe volume territories in winter and the dispersal of sons onto local breeding territories in spring.

Given the spatial dependence of mistletoe volume for distances up to 250 meters, the estimated mean winter territory radius of 115 meters, and data indicating that most dispersal events observed for sons fell within 0–250 meters of their natal nest, parental territories encompassing high quantities of mistletoe will typically have similarly high concentrations of mistletoe up to approximately 135 meters outside of their territory boundaries. This observed spatial autocorrelation of mistletoe volume supports the hypothesis that western bluebird sons remaining on winter territories with high mistletoe volume, as they tend to do [10], will acquire territories of similar quality when they settle next door and bud off a portion of their parents' territory in spring [9]. Specifically, using the ratio of correlated area (*A* = π250^2^) to estimated mean area over which mistletoe volume is spatially autocorrelated, we expect that between 2.8 and 5.2 additional territories could saturate a landscape within which spatial autocorrelation is still present. Most importantly, our results suggest that spatial autocorrelation of mistletoe volume is occurring at a scale relevant the persistent territoriality, winter family territoriality, and formation of kin neighborhoods in spring.

We cannot rule out the possibility that the underlying age structure, size or distribution of oak trees also play into the spatial aggregation of mistletoe. However, given that valley oaks are rare compared to blue oaks, it is unlikely that they generate the pattern of autocorrelation independently. Spatial autocorrelation of mistletoe volume could also be influenced disproportionately by relatively few, large plants, as opposed to many small plants, however, when large clumps of mistletoe are inspected very closely and pulled apart by cutting, it is clear that they often consist of multiple small plants that have grown together, a phenomenon that may reflect the favorite perch sites of birds living on territories.

The results of the Ripley's K and O-ring analyses provide evidence in favor of *some* seed dispersal occurring at greater scales up to 558 m. This pattern, while consistent with movements of cedar waxwings and American Robins, which are less common dispersers of mistletoe seeds on our study area, is also consistent with longer distance dispersal by western bluebirds, which make frequent trips off their territories to distant watering sites (J.L.D., unpublished observations). The observed repulsion detected by the O-ring analysis at scales beyond 558 m may be suggestive of competitive interactions, in which the likelihood of having nepotistic, related males living next door to each other decreases with distance, leading to an increased frequency of antagonistic interactions between adjacent nonrelatives.

Point data, defined loosely as presence or absence at a given location, are manifest as a non-continuous data set. However, the availability of continuous values (e.g. volume) associated with mistletoe points allowed us to examine the extent of spatial autocorrelation of resource abundance, which is more directly relevant to resource-dependent social behavior such as delayed dispersal and extreme philopatry of western bluebird sons. While the authors acknowledge that the interpretation of geostatistical methods applied to mistletoe point data should be viewed with caution, the availability of data on mistletoe volume provides an unusual opportunity to characterize some unequivocal characteristics of the underlying pattern of spatial autocorrelation.

Two complementary processes may be important to maintaining spatial autocorrelation: 1) long-term territory occupancy, in combination with territorial budding and local proliferation of dynasties from wealthy territories can increase local deposition and germination of mistletoe seeds around those territories, leading to increased spatial autocorrelation of mistletoe volume at scales of one to two territories and 2) local augmentation of mistletoe volume due to continuous occupancy can, in turn, increase the benefits of living in family groups, the likelihood that territories will be occupied for many years or even decades, and the frequency with which sons disperse next door to parents [9].

Although these relationships are usually viewed within the context of mutualism, persistent territoriality may not be beneficial to oak mistletoe if it reduces the potential for long-distance dispersal and colonization of new habitats or if it results in increased competition among mistletoe plants for germination sites or dispersal opportunities. This line of thinking is similar to recent conclusions drawn for the relationship between mistletoe and its specialist versus generalist mistletoe-eating birds [6], however, we specifically highlight territoriality and social behavior as fitness-related behaviors that can lead to patterns of seed dispersal that favor western bluebirds at the expense of oak mistletoe. Our evidence that mistletoe volume is associated with philopatry in western bluebirds is supported by both experimental and observational data [5], [9]. The spatial analysis in this paper is consistent with the idea that the underlying distribution of mistletoe is also influenced by the persistent territoriality of western bluebirds.
